# Phytochemical Fingerprinting and In Vitro Antimicrobial and Antioxidant Activity of the Aerial Parts of *Thymus marschallianus* Willd. and *Thymus seravschanicus* Klokov Growing Widely in Southern Kazakhstan

**DOI:** 10.3390/molecules26113193

**Published:** 2021-05-26

**Authors:** Bagda Sagynaikyzy Zhumakanova, Izabela Korona-Głowniak, Krystyna Skalicka-Woźniak, Agnieszka Ludwiczuk, Tomasz Baj, Krzysztof Kamil Wojtanowski, Aleksandra Józefczyk, Karlygash Altynbekovna Zhaparkulova, Zuriyadda Bektemirova Sakipova, Anna Malm

**Affiliations:** 1 Department of Pharmaceutical Technology, School of Pharmacy, Asfendiyarov Kazakh National Medical University, Tole Be Street 94, Almaty 050000, Kazakhstan; bagdush@mail.ru (B.S.Z.); zhaparkulova.k@kaznmu.kz (K.A.Z.); sakipova.z@kaznmu.kz (Z.B.S.); 2 Department of Pharmaceutical Microbiology, Faculty of Pharmacy, Medical University of Lublin, Chodźki Street 1, 20-093 Lublin, Poland; iza.glowniak@umlub.pl; 3 Independent Laboratory of Natural Products Chemistry, Department of Pharmacognosy, Faculty of Pharmacy, Medical University of Lublin, Chodźki Street 1, 20-093 Lublin, Poland; kskalicka@pharmacognosy.org (K.S.-W.); aludwiczuk@pharmacognosy.org (A.L.); 4 Department of Pharmacognosy, Faculty of Pharmacy, Medical University of Lublin, Chodźki Street 1, 20-093 Lublin, Poland; tbaj@pharmacognosy.org (T.B.); krzysztofkamilw@gmail.com (K.K.W.); ajozefczyk@pharmacognosy.org (A.J.)

**Keywords:** *Thymus* species, Kazakh flora, phytochemical analysis, antioxidant activity, antimicrobial activity, *Helicobacter pylori*

## Abstract

The chemical composition of the hydroethanolic extracts (60% *v/v*) from the aerial parts of *Thymus marschallianus* Willd (TM) and *Thymus seravschanicus* Klokov (TS) from Southern Kazakhstan flora was analyzed together with their hexane fractions. Determination of antibacterial, antifungal and antioxidant activities of both extracts was also performed. RP-HPLC/PDA and HPLC/ESI-QTOF-MS showed that there were some differences between the composition of both extracts. The most characteristic components of TM were rosmarinic acid, protocatechuic acid, luteolin 7-*O*-glucoside, and apigenin 7-*O*-glucuronide, while protocatechuic acid, luteolin 7-*O*-glucoside, luteolin 7-*O*-glucuronide, and eriodictyol predominated in TS. The content of polyplenols was higher in TS than in TM. The GC-MS analysis of the volatile fraction of both examined extracts revealed the presence of thymol and carvacrol. Additionally, sesquiterpenoids, fatty acids, and their ethyl esters were found in TM, and fatty acid methyl esters in TS. The antioxidant activity of both extracts was similar. The antibacterial activity of TS extract was somewhat higher than TM, while antifungal activity was the same. TS extract was the most active against *Helicobacter pylori* ATCC 43504 with MIC (minimal inhibitory concentration) = 0.625 mg/mL, exerting a bactericidal effect. The obtained data provide novel information about the phytochemistry of both thyme species and suggest new potential application of TS as a source of bioactive compounds, especially with anti-*H*. *pylori* activity.

## 1. Introduction

The plants from the *Lamiaceae*/*Labiatae* family, specifically the *Thymus* L. genus, comprised over 300 species distributed worldwide, including in Europe and Asia. They are known to contain two main classes of secondary metabolites, essential oils and polyphenols (flavonoids in particular). Such plants possess a wide spectrum of biological activities, including antioxidant, anti-inflammatory and antimicrobial actions. The extensive application of *Thymus* species in traditional medicine is based on their pharmacological properties and has been documented from ancient times. These plants have also been used as aromatic herbs and spices. Therefore, *Thymus* species are of great interest to the pharmaceutical, food, and cosmetic industries [1,2,3,4,5].

Over two thousand publications concerning the bioactivity of *Thymus* species and their applications for medicinal, culinary, and cosmetic purposes are available on PubMed (https://pubmed.ncbi.nlm.nih.gov/, accessed on 12 March 2021). The most known and extensively studied *Thymus* species is *T. vulgaris* L. [6,7]. In contrast, there are only a few publications concerning the chemical composition and antimicrobial (antibacterial/antifungal) activity of *Thymus marschallianus* Willd. [8,9,10,11] and *Thymus seravschanicus* Klokov [12,13]. The performed studies included the analysis of various plant extracts, as well as essential oils. The plant material used was derived mainly from the spontaneous flora of Moldavia, Uzbekistan, or China, and less often from botanical gardens.

The aim of the present work was to analyze the chemical composition of the hydroethanolic extracts obtained from the aerial parts of *T. marschallianus* and *T. seravschanicus* from the flora of Southern Kazakhstan, with the intent of providing novel information about the phytochemistry of both rarely studied thyme species. Additionally, the volatile compounds present in both extracts were assessed. Moreover, the determination of their antibacterial, antifungal, and antioxidant activities was also carried out to supplement the available literature data in this area.

## 2. Results

### 2.1. Phytochemical Analysis of the Hydroethanolic Extracts from the Aerial Parts of Thymus marschallianus and Thymus seravschanicus

The composition of hydroethanolic extracts from both *Thymus* species were analyzed using RP-HPLC/PDA and HPLC/ESI-QTOF-MS. The RP-HPLC/PDA method revealed that four phenolic acids (protocatechuic acid, caffeic acid, ferulic acid, rosmarinic acid) and five flavonoids (luteolin 7-*O*-rutinoside, luteolin 7-*O*-glucoside, apigenin 7-*O*-glucuronide, eriodictyol, naringenin) were identified in the hydroethanolic extracts from both *Thymus* species. Some compounds such as *p*-hydroxybenzoic acid, rosmarinic acid hydrate, and luteolin 7-*O*-glucuronide were found to be present additionally in *T. seravschanicus*, while luteolin was found only in *T. marschallianus*. An exemplary chromatogram is presented in Figure S1.

In order to confirm, but also to identify additional compounds in both analyzed extracts, an LC-MS analysis was performed. The HPLC/ESI-QTOF-MS results, including *m/z* values of molecular ions and fragments are presented in Table 1. In total, 21 compounds were identified in the extract of *T. marschallianus*. These were mainly phenolic acids and their derivatives, flavonoids and their glycosides, as well as organic acids and coumarins. In contrast, 15 compounds were identified in the extract of *T. seravschanicus*. These belong to the flavonoid and phenolic and organic acid groups. No coumarins were detected in this *Thymus* species. It is worth mentioning that the HPLC-PDA method only identified 10 compounds in *T. marschallianus* and 12 in *T. seravschanicus* (Figure S1).

The presence of rosmarinic acid was confirmed in both extracts. In the extract obtained from *T. seravschanicus*, the hydrate of this acid was additionally detected. Both species also produce caffeic, ferulic, and *p*-hydroxybenzoic acids. Moreover, 5-Methoxysalicylic acid was found in *T. seravschanicus*, while protocatechuic acid 3-*O*-glucoside was noted in *T. marschallianus*. In addition to the phenolic acids, quinic acid and quinic acid-sugar cluster (quinic acid + hexose_2_) were identified.

Eleven flavonoids belonging to the flavones subclass and two compounds from flavanones type were identified in the *T. marschallianus* extract. Among the flavones, luteolin and its glycosides, luteolin-7-*O*-rutinoside, luteolin-7-*O*-glucoside, luteolin-7-*O*-glucuronide, luteolin-7-*O*-dipentoside, and luteolin-7-*O*-(6″-3-hydroxy-3-methyl-glutaryl)-glucoside, apigenin and its glycosides, apigenin-7-*O*-glucoside, apigenin-7-*O*-glucuronide, and apigenin-7-*O*-rhamnoglucuronide, and diosmetin glucuronide were detected. Among the flavanones, the presence of eriodictyol and naringenin was confirmed. Regarding the flavonoids, only eight were detected in *T. seravschanicus*, while six of these eight compounds belonged to the flavone type and were identified as luteolin-7-*O*-rutinoside, luteolin-7-*O*-glucoside, luteolin-7-*O*-glucuronide, luteolin-7-*O*-(6″-3-hydroxy-3-methyl-glutaryl)-glucoside, apigenin-7-*O*-glucuronide, and diosmetin glucuronide. Eriodictyol and naringenin from the flavanone group were also detected.

A quantitative analysis of the phenolic compounds identified by the HPLC-PDA method was performed based on the external standard method. The data presented in Table 2 showed that among the phenolic acids, protocatechuic acid (2.08 ± 0.01 mg/g dry extract) was revealed to be dominant in *T. marschallianus*, while rosmarinic acid (3.33 ± 0.01 mg/g dry extract) and protocatechuic acid (3.06 ± 0.01 mg/g dry extract) were dominant in *T. seravschanicus*. A much higher flavonoids content was determined in *T. seravschanicus* than in *T. marschallianus*. Luteolin 7-*O*-glucoside (20.17 ± 0.12 mg/g dry extract), luteolin 7-*O*-glucuronide (16.84 ± 0.05 mg/g dry extract), and eriodictyol (13.54 ± 0.04 mg/g dry extract) were dominant in *T. seravschanicus*, while luteolin 7-*O*-glucoside (7.77 ± 0.14 mg/g dry extract) and apigenin 7-*O*-glucuronide (7.22 ± 0.01 mg/g dry extract) were dominant in *T. marschallianus*. Overall, the content of most compounds was higher in *T. seravschanicus* than in *T. marschallianus*—reaching values up to three times higher.

In addition to the polar components present in the hydroethanolic extracts, an analysis of the volatiles was also performed. To do this, the hexane fractions of both investigated thyme species extracts were obtained and analyzed by GC-MS. The results are shown in Table 3. In total, 14 and 15 compounds were identified in the hexane fractions of *T. marschallianus* and *T. seravschanicus* hydroethanolic extracts, respectively. We found that both *Thymus* species are characterized by the presence of two monoterpene alcohols: thymol and carvacrol. Other identified terpenoids were different in both species. Besides the two mentioned compounds, the *T. marshallianus* volatile fraction also contained several sesquiterpenoids (α-bisabolene, spathulenol, viridiflorol). Moreover, the presence of eugenol (a compound belonging to the phenylpropanoids) and quinic acid was confirmed. The latter compound was also detected by LC-MS analysis. The *T. seravschanicus* volatile fraction was composed mainly of monoterpenoids. Apart from the mentioned thymol and carvacrol, the presence of *p*-cymene, limonene, carvone, and thymoquinone weasrevealed. Moreover, the hexane fractions of both extracts were characterized by the presence of fatty acids and their esters. Hexadecanoic, linolenic, and α-linolenic acids and their ethyl esters were identified in *T. marschallianus*. In contrast, the methyl esters of the fatty acids were detected in the volatile fraction of *T. seravschanicus*.

### 2.2. The Total Polyphenol Content (TPC) in the Hydroethanolic Extracts from the Aerial Parts of Thymus marschallianus and Thymus seravschanicus, and Their Antimicrobial and Antioxidant Activity

**Figure 1:**
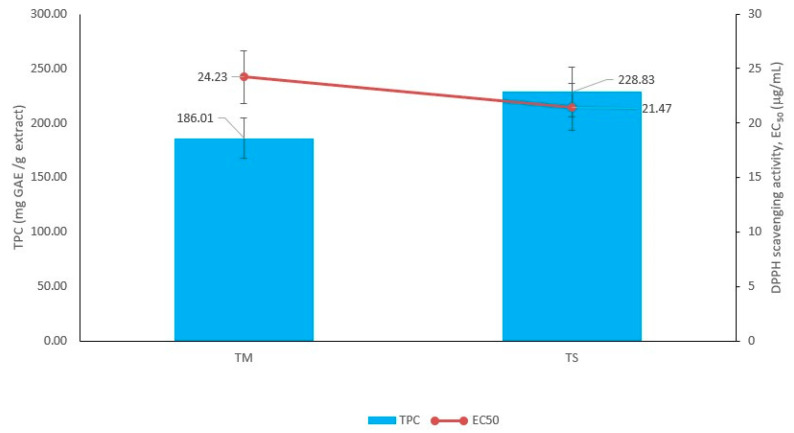
The total polyphenol content (TPC) in the hydroethanolic extracts from the aerial parts of *Thymus marschallianus* (TM) and *Thymus seravschanicus* (TS), together with their antioxidant activity. Mean values ± SD were presented (*n* = 3).

As presented in Figure 1, TPC, expressed as gallic acid equivalents (GAE), was somewhat higher in *T. seravschanicus* (228.83 ± 39.44 mg GAE/g extract) than in *T. marshallianus* (186.01 ± 16.11 mg GAE/g extract). Both extracts showed similar antioxidant properties to those assessed by DPPH scavenging activity; EC_50_ was 24.23 ± 0.29 μg/mL for *T. marshallianus* and 21.47 ± 1.63 μg/mL for *T. seravschanicus*. The antioxidant activity of both extracts determined by AAI (antioxidant activity index) was found to be 2.45 ± 0.03 for *T. marshallianus* and 2.78 ± 0.21 for *T. seravschanicus*.

As shown in Table 4, sensitivity of the reference strains of gram-positive and gram-negative bacteria to the hydroethanolic extracts from *T. marschallianus* and *T. seravschanicus* was found to differ depending on the extract, the group of bacteria, and the bacterial species. In general, extracts from *T. seravschanicus* was somewhat more active (MIC = 0.625–10 mg/mL) than those from *T. marschallianus* (MIC = 2.5–10 mg/mL). It should be noted that *T. seravschanicus* extracts were also more active against *H. pylori* ATCC 43504 (MIC = 0.625 mg/mL) than *T. marschallianus* extracts (MIC = 2,5 mg/mL). Both extracts possess bactericidal activity against the majority of bacterial species studied (MBC/MIC 1–2), except for *B*. *cereus* ATCC 10876 (MBC/MIC > 4). As presented in Table 4, both thyme extracts were found to exert the same activity against the reference strains of yeasts from the *Candida* genus with MIC = 5 mg/mL. The MFC for *C. albicans* ATCC 10231 was 10 mg/mL, while for *C. parapsilosis* ATCC 22019, this was 20 mg/mL. Both extracts possessed fungicidal activity with MFC/MIC = 2–4.

Apigenin and luteolin were used as the reference compounds in the present study. We saw great differences in the sensitivity to both flavonoids of the bacterial species studied (Table 4). The MIC of apigenin was 5–10 mg/mL for both Gram-positive and Gram-negative bacteria, except for *H. pylori* ATCC 43504, which was 1.25 mg/mL. In contrast, the MIC of luteolin varied from 0.156 to 1.25 mg/mL for Gram-positive bacteria and was the same, i.e., 10 mg/mL for Gram-negative bacteria, except for *H. pylori* ATCC 43504, which was 1.25 mg/mL. The yeast strains studied were found to possess differential sensitivity to both flavonoids, depending on the strain. *C. albicans* ATCC 102231 and *C. parapsilosis* ATCC 22019 were more sensitive to luteolin (MIC = 0.625 mg/mL) than to apigenin (MIC = 5 mg/mL). In contrast, *C. glabrata* ATCC 90030 showed greater sensitivity to apigenin (MIC = 0.625 mg/mL) than to luteolin (2.5 mg/mL).

## 3. Discussion

The flora of Southern Kazakhstan, not yet fully explored in terms of its chemical composition and biological activity, can be regarded as a rich reservoir of several medicinal plants with novel potential applications [14,15,16,17,18]. Phytochemical analysis of the hydroethanolic extracts obtained from the aerial parts of *T. marschallianus* Willd and *T. seravschanicus* Klokov growing widely in Southern Kazakhstan has indicated that there were several differences in the composition of the metabolites, as revealed by HPLC/ESI-QTOF-MS. The biggest difference is the presence of coumarins only in *T. marshallianus*. More compounds from the flavonoid group were detected in *T. marshallianus*, while more phenolic acids were identified in *T. seravschanicus*. The identified phenolic acids are very characteristic for all species classified in the *Lamiaceae* family. Among them, the most typical and, indeed, the chemical marker of the *Lamiaceae* family, is rosmarinic acid [19]. Quantitative analysis by RP-HPLC/PDA demonstrated that protocatechuic acid, luteolin 7-*O*-glucoside, and apigenin 7-*O*-glucuronide were dominant in the hydroethanolic extracts of *T. marschallianus*, while rosmarinic acid, protocatechuic acid, luteolin 7-*O*-glucoside, luteolin 7-*O*-glucuronide, and eriodictyol were dominant in those of *T. seravschanicus*. It should be noted that the content of phenolic acids and flavonoids mentioned above was mostly much higher in *T. seravschanicus* than in *T. marschallianus*.

The research conducted by Niculae et al. [11] using HPLC-DAD-ESI(+)-MS showed the presence of luteolin, quercetin, apigenin, and four of their derivatives (luteolin-7-*O*-glucuronide, quercetion-7-*O*-glucuronide, quercetin-7-*O*-arabinoside, apigenine-7-*O*-glucuronide) in the hydroethanolic (70% *v/v*) extract obtained from the aerial parts of *T. marschallianus* grown widely in North Eastern Moldavia. Additionally, they detected methyl-rosmarinate and rosmarinic acid as the most important compounds for this species. What is more, Mamadalieva et al. [12] performed studies on the phytochemical analysis of various extracts obtained from the aerial parts of *T. seravschanicus* from the Uzbek flora and identified two monoterpens (thymol, carvacrol) and three flavonoids (apigenin 7-*O*-glucoside, eriodictyol, naringenin) in the methanolic extract of this thyme species by the use of different chromatographic methods.

In case of the volatile fraction of the hydroethanolic extracts from the aerial parts of *T. marshallianus* and *T. seravschanicus*, the presence of thymol and carvacrol was additionally confirmed in both thyme species. The presence of sesquiterpenoids, fatty acids, and their ethyl esters were attested in *T. marshallianus*. Monoterpenoids and fatty acids methyl esters were the most characteristic volatiles for *T. seravschanicus*. As reported by Jia et al. [8], the major components of essential oil from *T. marschallianus* growing in China (Xinjiang) were found to be thymol, *p*-cymene and γ-terpinene among the 53 identified compounds. Moreover, these compounds, together with phellandral and thymol acetate, were the main components of the 63 compounds identified in the essential oil of *T. seravschanicus* from Uzbek flora [13].

The data presented in this work, resulting from the analysis of both hyroethanolic extracts using RP-HPLC/PDA, HPLC/ESI-QTOF-MS, and GC-MS methods, provide novel information about the chemical composition of both thyme species from the flora of Southern Kazakhstan.

Plants are valuable and rich sources of a wide range of secondary metabolites that possess multidirectional biological activity, including antioxidant, antibacterial, and antifungal antimicrobial properties [20]. Among these, the polyphenols are widely distributed [21,22,23,24]. The hydroethanolic extracts from the aerial parts of both thyme species studied demonstrated similar antioxidant activity when assessed by DPPH scavenging ability, and had almost similar TPC values. The antioxidant activity of both extracts determined by AAI was considered to be very strong. Sherer and Godoy [25] proposed a set of criteria for assessment of antioxidant activity based on AAI values. These are: weak (AAI < 0.5), moderate (AAI between 0.5 and 1.0), strong (AAI between 1.0 and 2.0), and very strong (AAI > 2.0).

According to the literature data [12], the antioxidant activity of the methanolic extract from *T. seravschanicus* from Uzbek flora was somewhat higher (IC_50_ = 15.87 0.44 μg/mL) than that presented here. In contrast to the data presented in this paper, antioxidant activity of the hydroethanolic (70% *v/v*) extract from *T. marschallianus* collected in North Eastern Moldavia was lower (IC_50_ = 81.2 ± 1.3 µg/mL) [26]. Moreover, as these authors were studying the effects of this lyophilized extract in Wistar rats with experimentally induced hyperglycemia, their results indicate that it exerted a beneficial effect by increasing the in vivo antioxidant capacity.

Our work demonstrates that the hydroethanolic extracts from the aerial parts of *T. marschallianus* and *T. seravschanicus* possess antibacterial and antifungal (anti-candidal) activity and show biocidal effect for most of the microbial species studied. It is generally stated that antimicrobials are usually regarded as bactericidal or fungicidal if the MBC/MIC or MFC/MIC ratio is ≤ 4 [27]. The observed somewhat higher antibacterial activity of *T. seravschanicus* in comparison to that from *T. marschallianus* may be due to higher content of flavonoids and phenolic acids in *T. seravschanicus*, but this requires further study. Overall, regarding the obtained MIC values for both thyme extracts, it should be noted that the MIC ≤ 1 mg/mL seen for both plant extracts may indicate their noteworthy antimicrobial (antibacterial) activity, and suggests that further research is needed [28]. However, Kuete and Efferth [29] proposed the following criteria to categorize the activity of plant extracts against bacteria and fungi: significant (MIC < 100 μg/mL), moderate (100 < MIC ≤ 625 μg/mL), or weak (MIC > 625 μg/mL). The observed somewhat higher antibacterial activity of *T. seravschanicus* in comparison to that from *T. marschallianus* may be due to a higher content of flavonoids, especially luteolin derivatives, in *T. seravschanicus*, but this requires further study.

Orłowska et al. [9] studied the antimicrobial activity of the methanolic extracts from 18 various thyme species growing in a botanical garden in Poland. They found using the dot blot test with direct bioautographic detection that *T. marschallianus* exterted considerably higher activity against Gram-positive bacterial species *Bacillus subtilis* ATCC 6633 than that of most herbs assayed. However, in these studies, *T. seravschanicus* was not included. In the present paper, Gram-positive bacteria were found to usually be more sensitive to both extracts as compared to those from Gram-negative bacteria. Both extracts showed mostly bactericidal action, except for their bacteriostatic activity against *B. cereus* ATCC 10876. These observations are in accordance with data presented by Niculae et al. [11]. They found higher activity of the *T. marschallianus* hydroethanolic (70% *v/v*) extract against *S. aureus*, *Staphylococcus pseudintermedius*, and *Bacillus cereus* (representatives of Gram-positive bacteria), in comparison to *Salmonella* Enteritidis, and no effect on *Salmonella* Typhimurium (both species belonging to Gram-negative bacteria). The antibacterial activity of *T. marschallianus* and *T. seravschanicus* essential oils was also reported elsewhere [13]. Moreover, as found by other authors [8], *T. marschallianus* and *T. seravschanicus* essential oils possessed activity against yeasts and/or molds.

The data presented in this paper indicate that the hydroethanolic extract from the aerial parts of *T. seravschanicus* was found to be the most active against *Helicobacter pylori* ATCC 43504 (MIC = 0.625 mg/mL), and exerted a bactericidal effect. These observations suggest a new potential application of *T. seravschanicus* as a source of bioactive compounds, especially with anti-*H*. *pylori* activity. It should be noted that *H. pylori* infection is the most common type found in the world’s population. Being of chronic character, this infection is involved in the development of peptic ulcer disease and gastric cancer. The resistance of *H. pylori* to antibiotics has recently reached alarming levels worldwide, which has a negative effect on treatment efficacy [30]. Past research reveals that many traditional medicinal plants are reported to possess promising anti-*H*. *pylori* activity in vitro and to show a potential as alternative and/or complementary candidates for *H. pylori* eradication and prevention of *H. pylori*-induced related gastric diseases [31].

## 4. Materials and Methods

### 4.1. Plant Material and Extraction Procedure

The aerial parts (herb) of *T. marschallianus* Willd. and *T. seravschanicus* Klokov were collected in April and May 2018 in the period before flowering at the foothills of the Kungei Alatau, Almaty region, Republic of Kazakhstan. The harvesting of *Thymus* herba was in accordance with the principles of the standards of Good Agriculture and Collection Practise (GACP) for Medicinal Plants. Raw material drying was carried out by the air-shadow method, in well-ventilated accommodations, out of direct sunlight. The collected raw materials of *T. marschallianus* Willd. (Voucher No. 01-07/301) and *T. seravschanicus* Klokov (Voucher No. 01-07/300) were identified in accordance with the requirements of the State Pharmacopoeia of the Republic of Kazakhstan, by Irina Otradnykh, senior scientist of the RSE Institute of Botany and Phytointroduction of Kazakhstan Academy of Sciences in Almaty, Kazakhstan. Raw materials of both *Thymus* species were dried at 35 °C to a moisture level below 10%. The degree of grinding of raw materials is 1.0–3.0 mm. The dried aerial parts (10 g) were then extracted twice with 60% (*v/v*) EtOH (2 × 200 mL) at 25 °C in an ultrasonic bath by ultrasound-assisted maceration (2 × 20 min). The combined extracts were filtered and evaporated to dryness in a vacuum evaporator at 40 °C.

### 4.2. Analysis of the Hydroethanolic Extracts by HPLC/ESI-QTOF-MS

The obtained extracts were analyzed qualitatively by an high-performance liquid chromatography–electrospray ionization-quadrupole-time of flight-mass spectrometry (HPLC/ESI-QTOF-MS) system in negative ion mode with use of 6530B Accurate-mass-QTOF-MS (Agilent Technologies, Inc., Santa Clara, CA, USA) mass spectrometer with an ESI-Jet Stream ion source. The Agilent 1260 chromatograph was equipped with DAD detector, autosampler, binary gradient pump, and column oven. Gradient of solvents: water with 0.1% formic acid (solvent A) and acetonitrile with 0.1% formic acid (solvent B) were used as the mobile phases. The following gradient procedure was adopted: 0–45 min, 0–60% of B; 45–46 min, 60–90% B; 46–50 min, 90% (B), the post time was 12 min. Total time of analysis was 57 min, with a stable flow rate of 0.200 mL/min. Injection volume for extracts was 10 μL. ESI- QTOF-MS analysis was performed according to the following parameters of the ion source: Dual spray jet stream ESI, positive and negative ion mode, gas (N2) flow rate: 12 L/min, nebulizer pressure: 35 psig, vaporizer temp.: 300 °C; *m/z* range 100–1000 mass units, with acquisition Mode Auto MS/MS, collision induced dissociation (CID): 20 eV with MS scan rate 1 spectrum per s, 2 spectra per cycle, skimmer: 65 V, fragmentor: 140 V and octopole RF Peak: 750 V. The tentative assignment of compounds was done on the basis of MS/MS spectra in the negative ion mode at CID energy of 20 eV. Identification was carried out by comparing the data with the literature and records included in the METLIN database [32].

### 4.3. Analysis of the Hydroethanolic Extracts by RP-HPLC/PDA

Before the analysis of polyphenolic constituents by high-performance reverse-phase liquid chromatography combined with a photodiode-array detection (RP-HPLC/PDA), dry extracts of both *Thymus* species were subjected to a purifying procedure using solid-phase extraction (SPE). For this purpose, a vacuum system (SPE-12G; J.T. Baker) connected to a vacuum pump (AGA-Labor, Warsaw, Poland) was used. Dry extract samples were dissolved with 2 mL of 80% (*v/v*) methanol and quantitatively transferred onto octadecyl BakerBond (500 mg, 3 mL) cartridges that had been activated previously with methanol (10 mL), followed by equilibration with 80% (*v/v*) methanol (10 mL). Extract samples were passed through the cartridges under vacuum. Interfering hydrophobic substances (e.g., chlorophyll) were retained in the solid phase and polyphenolic constituents were washed out with 80% (*v/v*) methanol directly into 10-mL calibrated flasks. The final concentration of all eluates obtained was 0.005 g of dry matter extract per 1 mL of sample solution. Next, an Agilent Technologies (Waldbronn, Germany) 1100 Series liquid chromatograph equipped with an autosampler and a PDA detector, set at 254 nm, 280 nm, or 325 nm was applied due to different maxima of absorbance of the analyzed polyphenols. Chromatographic separations were carried out on a Zorbax Eclipse XDB C8 column (150 × 4.6 mm I.D., dp = 5 μm) with a gradient elution: A—water + 1% (*v/v*) acetic acid; B—acetonitrile: 0 min—0% B; 0–10 min—10–14% B; 10–25 min—14–30% B; 25–35 min—30–35% B; 35–50 min—35–60% B; 50–57 min—60% B (isocratic) at a flow-rate of the mobile phase of 1 mL/min. A postrun time of 10 min was additionally introduced to the gradient program in order to equilibrate the chromatographic column before starting each new analysis. The temperature of the column compartment was maintained at 25 °C while performing the whole chromatographic separation. The linearity of the HPLC quantitative procedure for identified phenolic acids, their derivatives, and flavonoids was evaluated based on the external standard method. The solutions of reference compounds were prepared in 80% (*v/v*) methanol at five concentrations ranging from 0.02 to 0.1 mg/mL. Five-point curves were constructed and regression equations, together with *R*^2^ values, were calculated for each calibration curve. The chromatographic and spectroscopic data (peak areas corresponding to the concentrations of each quantified compound) were collected for inter-day (*n* = 3) analyses of both reference substance solutions and samples of *Thymus* extracts that were performed during the three consecutive days. The repeatability of peak areas was determined by the evaluation of SD. Mean values ± SD (standard deviation) were presented.

### 4.4. GC-MS Analysis of the Hexane Fractions of the Hydroethanolic Extracts

Dry extracts were shaken short with n-hexane in an ultrasonic bath at room temperature. Next, the hexane fractions were collected and analyzed by GC-MS. Analysis was performed with a Shimadzu GC-2010 Plus GC instrument coupled to a Shimadzu QP2010 Ultra mass spectrometer (Shim-Pol, Poland). Compounds were separated on a fused-silica capillary column ZB-5 MS (30 m, 0.25 mm i.d.) with a film thickness of 0.25 µm (Phenomenex). The following oven temperature program was initiated at 50 °C, held for 3 min, then increased to 250 °C at a rate of 5 °C/min, and held for 15 min. The spectrometers were operated in electron impact mode, the scan range was 40–500 amu, the ionization energy was 70 eV, and the scan rate was 0.20 s per scan. The injector, interface, and ion source were kept at 280, 250, and 220 °C, respectively. Split injection was conducted with a split ratio of 1:20 and helium was used as carrier gas at 1.0 mL/min flow rate. The retention indices were determined in relation to a homologous series of *n*-alkanes (C8–C24) under the same operating conditions. Compounds were identified using a computer-supported spectral library NIST 2011; MassFinder 2.1.

### 4.5. Determination of Total Polyphenol Content (TPC) in the Hydroethanolic Extracts

The total phenolic content in both *Thymus* extracts was determined spectrophotometrically by a modified method previously described by Clarke et al. [33] and Nickavar and Esbati [34]. Exactly 20 µL of extract dissolved in DMSO (conc. 10 mg/mL) and 100 µL of freshly prepared Folin-Ciocalteu reagent (diluted 1/10 with redistilled water) were added to the wells of a 96-well plate. After 5 min, 100 µL of a 7.5% Na_2_CO_3_ solution was added. The plates with the mixtures were incubated for 60 min at room temperature, then the absorbance was measured using an EPOCH spectrophotometer (Biotek, Winooski, VT, USA, Software ver. 3.08.01) at a wavelength of 760 nm. The same method was used to establish a calibration curve for the standard gallic acid (GAE) in the concentration ranges 7.5–120.0 µg mL^−1^ (y = 0.054 x + 0.029, *R*^2^ = 0,996). The analysis was performed in triplicate using DMSO as the blank. The content of total phenolic content expressed in equivalents as mg GAE/g of extract was calculated according to the formula described elsewhere [35]. Mean values ± SD were presented.

### 4.6. Determination of Antioxidant Activity of the Hydroethanolic Extracts

The antioxidant activity of both *Thymus* extracts was determined using the method described by Gai et al. [36] with modifications. Briefly, a starting solution was prepared by dissolving 10 mg of extract in 1 mL of DMSO solution. Then a series of dilutions were prepared in the same solvent at a concentration of 0.16–10 mg mL^−1^. Exactly 0.05 mL of each concentration was mixed with 0.15 mL of DPPH methanol solution (0.078 mg mL^−1^). The 96-well plate with the mixtures was incubated in the dark for 30 min at room temperature. Absorbance was measured at 515 nm (Biotek Epoch Microplate Spectrophotometer, Winooski, VT, USA, Software Version 3.08.01). The extract concentration needed to capture 50% of the initial DPPH (EC_50_) was determined automatically using 4-parameter logistic regression (4LP) from the plate reader software Gen5. The antioxidant activity was also expressed as the antioxidant activity index (AAI) determined according to the equation described elsewhere [25].

AAI = final concentration of DPPH (μg/mL)/EC_50_ (μg/mL) (1)

The experiments were performed in triplicate. Mean values ± SD were presented.

### 4.7. Determination of Antibacterial and Antifungal Activity of the Hydroethanolic Extracts

Both extracts were screened for antibacterial and antifungal activities by micro-dilution broth method according to the European Committee on Antimicrobial Susceptibility Testing (EUCAST) [37] using Mueller-Hinton broth and Mueller-Hinton broth with 5% lysed sheep blood for growth of non-fastidious and fastidious bacteria, respectively or RPMI with MOPS for growth of fungi. Minimal Inhibitory Concentration (MIC) of the tested extracts were evaluated for the panel of the reference microorganisms from American Type Culture Collection (ATCC), including Gram-negative bacteria (*Escherichia coli* ATCC 25922, *Salmonella* Typhimurium ATCC14028, *Klebsiella pneumoniae* ATCC 13883, *Pseudomonas aeruginosa* ATCC 9027, *Proteus mirabilis* ATCC 12453), Gram-positive bacteria (*Staphylococcus aureus* ATCC 25923, *Staphylococcus aureus* ATCC 6538, *Staphylococcus epidermidis* ATCC 12228, *Enterococcus faecalis* ATCC 29212, *Micrococcus luteus* ATCC 10240, *Bacillus subtilis* ATCC 6633, *Bacillus cereus* ATCC 10876), and fungi (*Candida albicans* ATCC 10231, *Candida parapsilosis* ATCC 22019). Minimal bactericidal concentration (MBC) or minimal fungicidal concentration (MFC) was also determined. The antimicrobial assays were performed as described previously [17,38]. *Helicobacter pylori* ATCC 43504, being a microaerophilic Gram-negative bacteria, was also included in these studies. MIC estimation was performed by micro-dilution broth method with resazurin as a growth indicator, as described previously [39]. Each experiment was repeated in triplicate. Of the three MIC and MBC or MFC values, the most common representative value, i.e., the mode, was presented.

## 5. Conclusions

Phytochemical analysis of the hydroethanolic extracts obtained from *T. marschallianus* and *T. seravschanicus* from the flora of Southern Kazakhstan revealed qualitative and quantitative differences in their chemical composition. However, both extracts showed similar antioxidant properties. It should be noted that *T. seravschanicus* possessed higher antibacterial activity, especially against *H. pylori*. The obtained novel data suggest the potential application of both thyme species as a source of bioactive compounds, especially *T. seravschanicus* with its anti-*H. pylori* activity. However, further studies are needed to confirm and to verify the obtained results using clinical isolates of *H. pylori* and to identify the compounds responsible for this activity.

## Figures and Tables

**Table 1:** Results of the HPLC/ESI-QTOF-MS analysis of the hydroethanolic extracts from the aerial parts of *T. marschallianus* (TM) and *T. seravschanicus* (TS).

No	Tentative Assignment	t_R_ [min]	Formula	Molecular Ion [*m/z*]	MS/MS Fragments [*m/z*]	TM	TS
Organic acids							
1	Quinic acid + hexose_2_	1.594	C_19_H_34_O_17_	533.1738	191.0577	+	+
2	Quinic acid	2.000	C_7_H_12_O_6_	191.0577	179.0577	+	+
Phenolic acids							
3	Protocatechuic acid 3-*O*-glucoside	6.906	C_13_H_16_O_9_	315.0727	153.0108; 109.0290	+	-
4	Hydroxybenzoic acid	9.885	C_7_H_6_O3	137.0249	109.0311	+	+
5	Caffeic acid	17.440	C_9_H_8_O_4_	179.0350	135.0437	+	+
6	5-Methoxysalicyllic acid	19.207	C_8_H_8_O_4_	167.0350	108.0217	-	+
7	Rosmarinic acid hydrate	19.897	C_18_H_18_O_9_	377.0878	359.0761; 197.0455; 161.0244	-	+
8	Ferulic acid	25.803	C_10_H_10_O_4_	193.0516	133.0322; 115.0209	+	+
9	Rosmarinic acid	27.082	C_18_H_16_O_8_	359.0780	179.0431; 161.0311; 133.0317	+	+
Flavonoids							
10	Luteolin 7-*O*-rutinoside	23.493	C_27_H_30_O_15_	593.1512	285.0412; 133.0255	+	+
11	Luteolin 7-*O*-glucoside	24.233	C_21_H_20_O_11_	447.0937	285.0405; 133.0289	+	+
12	Luteolin 7-*O*-glucuronide	25.252	C_21_H_18_O_12_	461.0743	285.0407; 133.0279	+	+
13	Apigenin 7-*O*-glucoside	25.986	C_21_H_20_O_10_	431.0991	268.0402; 151.0024	+	-
14	Luteolin 7-*O*-dipentoside	26.396	C_25_H_26_O_14_	549.1267	418.1281; 285.0578	+	-
15	Apigenin 7-*O*-glucuronide	27.440	C_21_H_18_O_11_	445.0781	269.0459; 135.0431; 117.0320	+	+
16	Diosmetin glucuronide	27.909	C_22_H_20_O_12_	475.0885	299.0519; 284.0310; 255.0179; 151.0052; 133.0259	+	+
17	Luteolin-7-*O*-(6″-3-hydroxy-3-methyl-glutaryl)-glucoside	28.386	C_27_H_28_O_15_	591.1355	549.1358; 531.1091; 285.0405	+	+
18	Eriodictyol	30.001	C_15_H_12_O_6_	287.0579	151.0035; 135.0439	+	+
19	Apigenin 7-*O*-rhamnoglucuronide	30.551	C_27_H_28_O_14_	575.1406	515.1170; 269.0467	+	-
20	Luteolin	31.453	C_15_H_10_O_6_	285.0408	133.0290; 107.0119	+	-
21	Naringenin	33.346	C_15_H_12_O_5_	271.0617	151.0037; 119.0536	+	+
22	Apigenin	34.269	C_15_H_10_O_5_	269.0462	135.0432; 117.0335	+	-
Coumarins							
23	Umbeliferone derivative 1	33.598	C_17_H_14_O_6_	313.0722	161.0250; 133.0310; 105.0347	+	-
24	Umbeliferone derivative 2	34.294	C_17_H_14_O_6_	313.0722	161.0200; 133.0284; 105.0331	+	-

(+)—present; (-)—absent.

**Table 2:** The content of polyphenols in the hydroethanolic extracts from the aerial parts of *Thymus marschallianus* (TM) and *Thymus seravschanicus* (TS).

No	Identified Compounds	Content (mg/g Dry Extract)	
		TM	TS
1	protocatechuic acid	2.08 ± 0.01	3.06 ± 0.01
2	*p*-hydroxybenzoic acid	nd	0.16 ± 0.00
3	caffeic acid	1.08 ± 0.01	1.92 ± 0.01
4	ferulic acid	0.13 ± 0.00	0.18 ± 0.01
5	rosmarinic acid hydrate	nd	1.29 ± 0.01
6	rosmarinic acid	1.05 ± 0.01	3.33 ± 0.01
7	luteolin 7-*O*-rutinoside	0.62 ± 0.01	2.10 ± 0.01
8	luteolin 7-*O*-glucoside	7.77 ± 0.14	20.17 ± 0.12
9	luteolin 7-*O*-glucuronide	nd	16.84 ± 0.05
10	apigenin-7-*O*-glucuronide	7.22 ± 0.01	6.81 ± 0.06
11	eriodictyol	2.29 ± 0.02	13.54 ± 0.4
12	luteolin	0.17 ± 0.01	nd
13	naringenin	0.32 ± 0.01	0.28 ± 0.01

Mean values ± SD were presented (*n* = 3).

**Table 3:** Comparison of the volatile compounds occurring in the hexane fractions of hydroethanolic extracts of the aerial parts of *Thymus marschallianus* (TM) and *Thymus seravschanicus* (TS).

No.	Identified Compounds	RI^a^	RI^b^	TM	TS
1	*p*-Cymene	1023	1015		+
2	Limonene	1027	1025		+
3	Carvone	1244	1243		+
4	Thymoquinone	1250	1249		+
5	Thymol	1296	1267	++	++
6	Carvacrol	1304	1278	+	+
7	Eugenol	1351	1331	+	
8	α-Bisabolene	1505	1503	+	
9	Spathulenol	1578	1572	+	
10	Viridiflorol	1606	1592	+	
11	Methyl hexadecanoate	1920	1909		++
12	Hexadecanoic acid	1961	1951	++	++
13	Ethyl hexadecanoate	1987	1978	+	+
14	Methyl linoleate	2086	2066		+
15	Methyl α-linolenate	2092	2092		++
16	Phytol	2103	2099	+	+
17	Linoleic acid	2131	2130	+	+
18	α-Linolenic acid	2138	2113	++	++
19	Ethyl linoleate	2152	2153	+	
20	Ethyl α-linolenate	2158	2166	+	
21	Stearic acid	2162	2174		+

RI*^a^*—retention index on HP-5MS column; RI*^b^*—retention index based on the data available in from Mass Finder 2.1 and NIST 2011 libraries. The relative content of identified compounds was indicated as high (++) and low (+) based on the peak’s surface area in corresponding chromatograms.

**Table 4:** Antimicrobial activity in vitro of the hydroethanolic extracts from the aerial parts of *Thymus marschallianus* (TM) and *Thymus seravschanicus* (TS).

Microbial Species	TM		TS		Apigenin	Luteolin
	MIC ^1^ (mg/mL)	MBC ^2^ or MFC ^3^ (mg/mL)	MIC (mg/mL)	MBC or MFC (mg/mL)	MIC (mg/mL)	MIC (mg/mL)
Antibacterial activity						
*Staphylococcus aureus* ATCC 25923	2.5	5	1.25	2.5	10	0.625
*Staphylococcus epidermidis* ATCC 12228	5	5	1.25	2.5	10	0.156
*Enterococcus faecalis* ATCC 29212	20	20	2.5	2.5	10	0.625
*Micrococcus luteus* ATCC 10240	5	10	2.5	2.5	10	0.313
*Bacillus subtilis* ATCC 6633	5	20	5	10	10	1.25
*Bacillus cereus* ATCC 10876	5	>20	2.5	>20	10	0.625
*Salmonella* Typhimurium ATCC 14028	10	20	10	10	10	10
*Escherichia coli* ATCC 25922	10	10	10	10	10	10
*Proteus mirabilis* ATCC 12453	10	10	2.5	5	10	10
*Klebsiella pneumoniae* ATCC 13883	5	5	1.25	5	10	10
*Pseudomonas aeruginosa* ATCC 9027	10	10	5	10	10	10
*Helicobacter pylori* ATCC 43504	2.5	2.5	0.625	0.625	1.25	0.625
Antifungal activity						
*Candida albicans* ATCC 102231	5	10	5	10	5	0.625
*Candida parapsilosis* ATCC 22019	5	20	5	10	5	0.625
*Candida glabrata* ATCC 90030	5	10	5	10	0.625	2.5

^1^ MIC (minimal inhibitory concentration), ^2^ MBC (minimal bactericidal concentration), ^3^ MFC (minimal fungicidal concentration). The representative values (mode) were presented (*n* = 3).

## Data Availability

All data are included in the article.

## Supplementary Materials

The following are available online. Figure S1. RP-HPLC/PDA chromatogram (at 325 nm) of the hydroethanolic extract from the aerial parts of *Thymus marschallianus* and *Thymus seravschanicus*.

## Sample Availability

Samples of the dry plant material are available from the authors at Asfendiyarov Kazakh National Medical University, School of Pharmacy, Almaty, Kazakhstan.
