# Supplementary material for: Phytochemical Fingerprinting and In Vitro Antimicrobial and Antioxidant Activity of the Aerial Parts of Thymus marschallianus Willd. and Thymus seravschanicus Klokov Growing Widely in Southern Kazakhstan
Source: Molecules. 2021 May 26;26(11):3193. doi: 10.3390/molecules26113193 (PMC8198081; doi:10.3390/molecules26113193)
Supplement: Supplementary file 1 [file molecules-26-03193-s001.zip › molecules-1212149-supplementary.pdf]

molecules

Supplementary Materials

Phytochemical Fingerprinting and In Vitro Antimicrobial and Antioxidant Activity of the Aerial Parts of *Thymus marschallianus* Willd. and *Thymus seravschanicus* Klokov Growing Widely in Southern Kazakhstan

Bagda Sagynaikyzy Zhumakanova, Izabela Korona-Głowniak, Krystyna Skalicka-Woźniak, Agnieszka Ludwiczuk, Tomasz Baj, Krzysztof Kamil Wojtanowski, Aleksandra Józefczyk, Karlygash Altynbekovna Zhaparkulova, Zuriyadda Bektemirova Sakipova and Anna Malm *

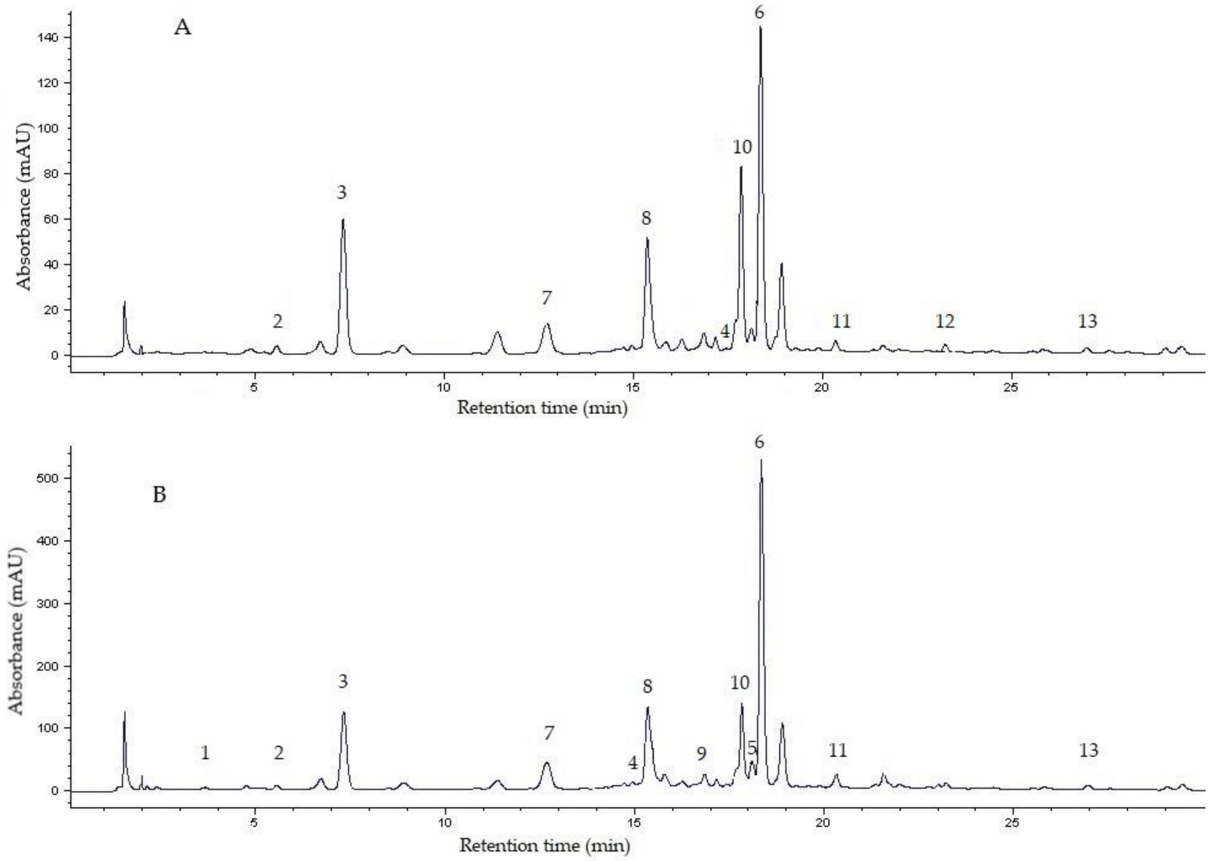

Figure S1. RP-HPLC/PDA chromatogram (at 325 nm) of the hydroethanolic extract from the aerial parts of *Thymus marschallianus* (**A**) *and Thymus seravschanicus* (**B**). Legend: protocatechuic acid (1), *p*-hydroxybenzoic acid (2), caffeic acid (3), ferulic acid (4), rosmarinic acid hydrate (5), rosmarinic acid (6), luteolin 7-O-rutinoside (7), luteolin 7-O-glucoside (8), luteolin 7-O-glucuronide (9), apigenin 7-O-glucuronide (10), eriodictyol (11), luteolin (12), naringenin (13).
